# Design of optimized photonic-structure and analysis of adding a SiO_2_ layer on the parallel CH_3_NH_3_PbI_3_/CH_3_NH_3_SnI_3_ perovskite solar cells

**DOI:** 10.1038/s41598-023-43137-3

**Published:** 2023-09-23

**Authors:** Mohammad Hosein Mohammadi, Mehdi Eskandari, Davood Fathi

**Affiliations:** 1 Department of Electrical and Computer Engineering, Tarbiat Modares University (TMU), Tehran, Iran; 2 Nanomaterial Research Group, Academic Center for Education, Culture and Research (ACECR) on TMU, Tehran, Iran

**Keywords:** Solar cells, Optical materials and structures, Solar cells

## Abstract

So far, remarkable achievements have been obtained by optimizing the device architecture and modeling of solar cells is a precious and very effective way to comprehend a better description of the physical mechanisms in solar cells. As a result, this study has inspected two-dimensional simulation of perovskite solar cells (PSCs) to achieve a precise model. The solution which has been employed is based on the finite element method (FEM). First, the periodically light trapping (LT) structure has been replaced with a planar structure. Due to that, the power conversion efficiency (PCE) of PSC was obtained at 14.85%. Then, the effect of adding an SiO_2_ layer to the LT structure as an anti-reflector layer was investigated. Moreover, increasing the PCE of these types of solar cells, a new structure including a layer of CH_3_NH_3_SnI_3_ as an absorber layer was added to the structure of PSCs in this study, which resulted in 25.63 mA/cm^2^ short circuit current (J_sc_), 0.96 V open circuit voltage (V_oc_), and 20.48% PCE.

## Introduction

The photovoltaic effect is the process by which light is converted into electricity. The first solid state solar cell was built in 1883 by Charles Fritts by melting a thin plate of selenium between gold and another metal as a conductor^1, 2^. Russell OHL first used silicon in 1939 to make solar cells^3,4^. But in general, the light absorption of silicon cells is relatively poor. Therefore, to use this material in the construction of solar cells, a wafer thickness of about 150 um is needed to absorb the maximum amount of sunlight. Hence, according to recent advances, scientists have obtained PSCs^5^. The first examples of perovskites, mineral compounds with the formula CaTiO_3_, were discovered in 1831 by a Russian scientist, whose structure is one of the three structures of materials in the form of cubes with the general formula ABX_3_. The most common form is that A is at the center of the cube and B ions are at the corners of the cube surrounded by 6 negative X ions in an octahedral fashion^6,7^. Materials with a perovskite structure have different properties that can change under different temperature and pressure conditions. Also, by changing each of the atom’s A, B, or X, we see that the properties of the material change. Organo metal halide is a group of perovskite structures that are used as light-absorbing layers in solar cells by replacing A with an organic cation, B with a metal-cation, and X with halides^8^. CH_3_NH_3_ can be used instead of A. Also, due to the bonds between the molecules, divalent metals are the best choice for B, and among them, Pb and Sn, due to their suitable optoelectronic properties, the need for low temperature for fabrication, and abundance in the earth's crust, have been used. Also, the most commonly used halogens include Cl and I. The most common perovskite materials used in solar cells today are CH_3_NH_3_BX_3_^9–11^. The advantages of perovskite materials include high absorption coefficient, high dielectric coefficient, very high mobility of carriers, high stability and efficiency, and a low-cost manufacturing process. But these perovskite materials also have disadvantages, including ferroelectric properties, their departure from the perovskite state when exposed to moisture or UV waves, and the presence of Pb in the perovskite structure, which is a toxic substance^12–15^.

The structure of PSCs is generally a light-absorbing perovskite, two layers of electron and hole transfer material (ETM and HTM) of organic or inorganic on either side of the perovskite, and metal contacts (usually ITO or FTO with low electrical resistance and high optical conductivity) at the top or bottom of the cell^16–18^. The basis of the work of PSCs is that sunlight strikes the perovskite material and is absorbed by it due to the used perovskite band gap. Due to the absorption of light in the perovskite, excitons are generated and, under the influence of the electric field, the excitons are decomposed into electrons and holes. Due to their mobility, these carriers move in the perovskite and enter the ETL (electron transfer layer) and HTL (hole transfer layer), and then transfer through these layers to metal contacts and generate electric current^19–24^. PSCs have been significantly welcomed by scientists over the past few years because of the benefits mentioned. Thus, the power conversion efficiency (PCE) of these cells has increased from 3.8% in 2009 to 18.4% in 2019. This improvement process is still increasing with the use of LT techniques and optimizations of nanostructures^25–31^.

The most accepted way to achieve light trapping (LT) in thin-film solar cells is to exploit the high refractive index guiding properties of the constituent materials.^32,33^. In this study^32^, the slotted and inverted prism structured SiO2 layers are adopted to trap lighter into the solar cells, and a better transparent conducting oxide layer is employed to reduce the parasitic absorption and with this strategy they could approach to an impressive value of 21.16%, being 31.2% larger than that for the solar cell without light management. The majority of efforts have been put towards creating transparent interfaces like thin nanowires, Au electrodes, transparent metal oxides, and transparent polymers in order to increase the transparency of PSCs^34,35^. Relatively little effort has been put into the construction of the perovskite photo absorber layer itself. To completely absorb photons with excitation energies higher than the bandgap of the perovskite photo absorber, a film thickness of at minimum 400 nm is necessary^36,37^. In the reference of^36^. An theoretical and experimental study of a PSC with the structure b-i-p was carried out. An efficiency of 13.15% was obtained experimentally with CH_3_NH_3_PbI_3_ and Spiro-OMeTAD thicknesses of 300 and 213 nm, respectively. Using the simulation tools, an optimization of thickness was performed, predicting an efficiency of 15.50% with 400 nm of CH_3_NH_3_PbI_3_ and 100 nm of spiro-OMeTAD. In contrast, expanding the thickness of the absorbing layer to increase efficiency reduces the transparency of the cell. On the other hand, as was previously shown in attempts to build ultrathin PSCs^38^, lowering the thickness of the perovskite light absorption layer to improve transparency affects efficiency. Using LT perovskite thin films that may concurrently increase optical absorption and transmittance over the wavelength range of perovskite optical absorbers is a viable way to solve this conundrum. PSC s have undergone a number of experiments to improve their light-harvesting abilities, and certain light-emitting layers created for dye solar cells have also been employed. In conclusion, light scattering by lengthening the light path of incoming light and light matrix paired via surface plasmon resonance are two typical methods of light absorption with light absorbers^39–41^. Pascoe et al.^40^ a textured CH_3_NH_3_PbI_3_ morphology formed through a thin mesoporous TiO_2_ seeding layer and a gas-assisted crystallization method have reported. The textured morphology comprises a multitiered nanostructure, which allows for significant improvements in the light harvesting and charge extraction performance of the solar cells. average J_sc_ for device was in excess of 22 mA/cm^2^, and the maximum recorded PCE was 16.3%. Utilizing textured perovskite light absorbers, in particular, can improve dispersion in PSCs while simultaneously facilitating charge carrier movement by increasing the area of contact between both the perovskite layer (absorber) and the charge collection layer^42^. In the reference^42^, Ullah et al. using controllable photonic structures and plasmonic nanoparticles in PSCs significantly affect the PCE. The simulation results showed that using a photonic structure significantly affects the photovoltaic result of the PSC, and the PCE reached from 15.62% for the planar structure to 16.17% for the photonic structure. Yet, it is challenging to produce such structures directly. Although constructing such a framework can be challenging, the PCE of the PSC will benefit greatly once it is completed. Yet, it is challenging to produce such structures directly. Although constructing such a framework can be challenging, the PCE of the PSC will benefit greatly once it is completed. In recent years, various LT approaches for PSC have been proposed, such as nano-cone arrays^43–45^, fiber anti-reflection front devices^46,47^, nano-pyramids^48–50^, nanophotonic^31,51^, nano-rod based on charge transport layers^24^, and corrugated substrates in PSCs^52^, as well as the exploitation of surface plasmon resonances, e.g., plasmonic nanoparticles^53–56^. To have strong absorption in the absorbent layer, the thickness of this layer must be increased. This increase has negative effects on the electrical properties of the PSC. Using periodic arrays of nanostructures that lead to LT in the PSC is an effective way to improve the absorption rate inside the active layer. Finally, a new research window opens in the process of improving the PCE of this type of cell^57^. The basis of LT structures is based on anti-reflective and light scattering effects, which can be very effective in improving absorption^52,58^. Therefore, many methods have been used to increase efficiency in PSCs, all of which mainly affect the electrical properties. Hence, with a light management strategy, the optical part of PSCs can also be optimized^59^. The output current is directly related to the absorption of the active layer, which normally absorbs about 65% of the incoming light. This means that the incoming light is lost by about 2% by ETL, 14% by the ITO layer, 4% by the glass surface, and 15% reflected from the surface of the device. Consequently, the use of an anti-reflective layer and optimal structure can be very effective in reducing light loss^43,60–63^.

To reach record performance, metal-halide PSCs must effectively control light. To improve the J_sc_ for high energy yields, criteria on materials, and photonic engineering are defined in this article. In order to further improve performance in the case of all-perovskite, two-terminal parallel cells, a perspective obtained from meticulous electromagnetic simulations done on several comparable structures is also introduced. In reference^64^, it is effective to boost absorption and decrease energy losses by utilizing light-trapping nanostructures and activating the device's plasmonic. In order to limit light in the active layer and improve energy harvesting, a unique configuration of a nanostructured PSC with a plasmonic enhancement has been developed in this article. Calculations show that the improved arrangement provides a 21% increase in PCE and a 23.4% increase in J_sc_ over the conventional PSC. In the experimental work, Wang et al.^65^ reported the high PCE using LT structure in PSC. The isopropanol (IPA)—assisted recrystallizing process allows for the formation of pinhole-free perovskite films on a substrate with a high aspect ratio, relaxing the trade-off between optical improvements and electrical deterioration. A modest 200 nm of MAPbI_3_ was used as the absorber in the PSC, which results in an efficiency of 18.6%, a record for such thin-PSCs and also, they displayed solar cells that, when compared to conventional planar devices, an increase in daily produced power of 47.6% due to the use of crater-like architecture. In the modeling work^66^, For traditional PSCs, the thickness of ITO layer and the perovskite layer were both first tuned and a 22.4% improvement in light absorption is possible. Then, using hemisphere, cylinder, inverted pyramid, and cone structures, this reference proposed four different types of multilayer conformal structures PSCs. The optimized hemispherical multilayer conformal structure (HMCS) PSCs may further increase light absorption by 12.3% when compared to the original PSCs and achieve the greatest photocurrent density of 23.82 mA/cm^2^. Haque et al.^67^ have designed an innovative industrially appealing paradigm for solar cells that achieves light trapping by conformably depositing the solar cell components onto photonic substrates that have been previously designed. In order to increase flexibility, this solution was used and optimized for PSCs with different perovskite absorber thicknesses, including conventional (500 nm) and ultra-thin (300 nm). This results in J_sc_ improvements of up to 22.8% in superstrate cell configuration and 24.4% in substrate-type configuration.

Another important factor to consider for perovskite materials is the energy gap. This is because perovskites act as light absorbers in solar cells, and therefore their energy gap affects the amount of light, or the wavelength range absorbed by them. Experimental and computational studies show that CH_3_NH_3_PbI_3_ has a direct band gap in all phases, and the band gap is equal to 1.55 eV^68,69^. Therefore, to increase the absorption spectrum in PSCs, other perovskite materials that have a smaller band gap can be used. Because of the smaller band gap, low-energy photons can be absorbed. In order to use all the energy of sunlight, perovskite materials can be used in tandem in the structure of PSCs. On the other hand, it is necessary to state here that because Pb is a toxic and dangerous material, studies have been done to replace it with Sn. According to experimental studies, the MASnI_3_ band gap is measured at about 1.3 eV, which reduces the band gap and increases its absorption range^70–74^. In^75^ a new structure is introduced which includes a layer of CH_3_NH_3_SnI_3_ as a second absorber layer in the PSC structure based on CH_3_NH_3_PbI_3_. In this study^75^ a layer of CH_3_NH_3_SnI_3_ with 100 nm thickness was added to the CH_3_NH_3_PbI_3_ PSC structure. Due to the lower band-gap of CH_3_NH_3_SnI_3_ compared to the CH_3_NH_3_PbI_3_, photons with lower energy can be absorbed in this layer and power conversion efficiency (PCE) was increased from 14.32 to 15.32% when the thickness of CH_3_NH_3_SnI_3_ is 200 nm.

Researchers have employed a variety of techniques, including changing the perovskite absorbent^76^, altering the side layer materials to accommodate the energy band levels^77^, modifying the layer doping and thickness, interface engineering^78,79^, modifying the device architecture^80^, and others. One of them that has received less attention is the device design and alteration of cell structure. The configuration of the cells with the same density of materials without changing the arrangement of layers or substances presents the most engineering problem in this method. Through the light-trapping mechanism, modifications to the architecture of solar cells and the use of nanostructures in their design may increase their efficiency. The emitted light into the cell will not be completely absorbed, and a sizeable percentage of it will be squandered, despite high-precision lithography, designed interfaces, and well-adjusted energy bands, as we will explore later in this study. This situation is caused by two factors: first, the PSC surface's reflection prevents light from entering the cell entirely; and second, sunlight energy is not only absorbed by the active layer but also by the inactive layers. Numerous nanostructures have been developed for solar cells to trap light in the absorbing layer in order to solve these problems. The objective of this work is to control the path of light inside the cell so that it stays inside the active layer.

**Figure 1:**
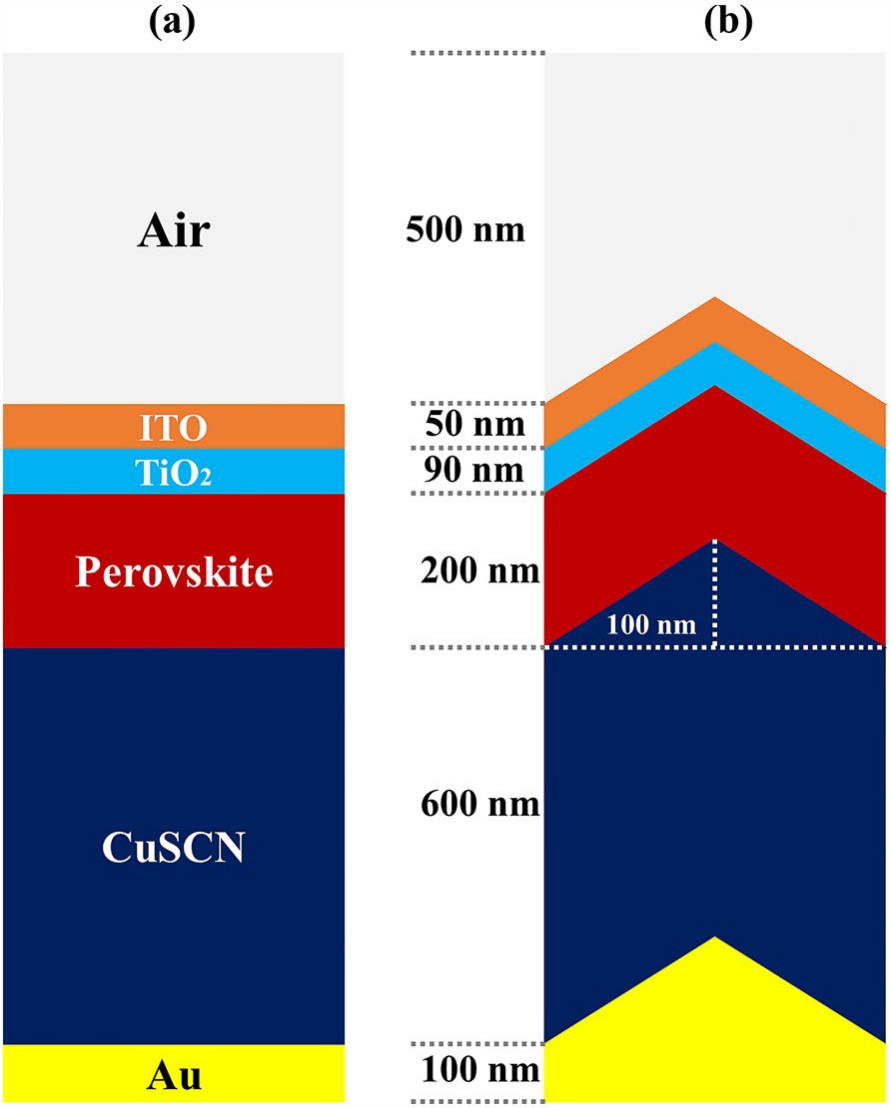
Schematic of (**a**) PSC with a planar structure and (**b**) a proposed PSC with a LT structure.

In this study, an optimal and useful method for the structure of PSCs has been implemented that can help improve the optical part. An LT structure is studied for all layers in the PSC as shown in Fig. 1. This figure shows two planes and one LT structure along with the layer thickness. This method has significant practical advantages in terms of its application compared to the planar structure, and helps to trap light in the whole structure. This structure is not only useful for PSCs but can also be used in other generations of solar cells such as silicon, thin-film, etc. Next, to prevent light loss at the beginning of the entry, we placed a SiO_2_ layer in the form of an LT structure on top of the ITO layer and examined the reflection results. Finally, to achieve maximum PCE in this type of structure, a new layer of perovskite (CH_3_NH_3_SnI_3_) material was used as a complement in absorption.

Micro and nanostructures can be shaped using a variety of techniques and are utilized in nano devices, micro actuators, and micro sensors. In order to form the described light trapping structure, the layers of this cell must be placed on a textured silicon substrate. Contrary to planar structures, this structured solar cell should be constructed starting from the bottom layer.

In recent decades, silicon micro-machining technology have achieved significant advancements^81^. This is because silicon has valuable qualities such the ability to be accurately shaped, compatibility with processes required to make integrated circuits, wide availability, and acceptable electrical, mechanical, and thermal properties^82^. As a result, the creation of nanostructured solar cells might likewise utilize these cutting-edge technologies.

This article is organized into 4 sections. The first part introduces the evolution of perovskite materials and how they are used in PSCs, as well as how LT structures function in solar cells. In the second part, the proposed PSC simulation and important parameters in the simulation are given. In the third part, the simulation results are examined in detail, and in the final part, the conclusion has been collected.

## Methods

### Theory

For an accurate simulation, we used two models: optical and electrical, and we will explain these two models in detail.

#### Optical model

In order to have a detailed analysis of how the LT structure works, we obtained the electric field in the whole structure using Maxwell equations:

1 $$\frac{\partial \mathrm{H}}{\partial \mathrm{t}}=\frac{-1}{\mu }\nabla \times E,$$

2 $$\varepsilon \frac{\partial E}{\partial t}=-\nabla \times H-\sigma E,$$

where H and E are the magnetic field intensity, E and electric field intensity respectively, µ is the permeability, σ is the electric conductivity and ε is the permittivity. Now, using Maxwell's equations and Eq. (3), the generation rate of carriers in the active layer can be obtained^83^:

3 $${G}_{OPT}=\frac{\varepsilon "{E}^{2}}{2\hslash }.$$

where E is the electric field, h is the Plank’s constant, and $\varepsilon "$ is the imaginary part of the relative permittivity defined as *ɛ*_r_ (λ) = (n(λ) − ik(λ))^2^ which is a function of the wavelength of propagation light. As it is clear, in order to increase ${G}_{OPT}$, we must increase the amount of E, which we intend to achieve with the LT structure.

#### Electrical model

After obtaining the carrier generation rate in the optical model, the current–voltage characteristic can now be obtained. For this purpose, we apply the output of the optical model results as input parameters to the electrical model. The following equation can be used to obtain the current–voltage characteristic of each solar cell:

4 $$J\left(V\right)={J}_{dark}-{J}_{sc}={J}_{0}\left(exp\left(\frac{eV}{nKT}\right)-1\right)-q{G}_{OPT}\left({L}_{n}+{L}_{p}\right)$$

*J*_*dark*_ is the solar cell current when no carrier generation. To obtain the short circuit current (J_sc_), the Poisson and continuity equations must be solved in PSC, for this purpose:

5 $$\nabla .\left({\varepsilon }_{0}.{\varepsilon }_{r}\nabla \varphi \right)=-\rho ,$$

6 $$\frac{\partial n}{\partial t}=\frac{1}{q}\nabla {j}_{n}+{G}_{n}-{U}_{N},$$

7 $$\frac{\partial p}{\partial t}=\frac{1}{q}\nabla {j}_{p}+{G}_{p}-{U}_{p},$$

where φ, q, ε_0_ and ρ are the electrostatic potential, charge of electron, vacuum permittivity and charge density respectively. U_N_ is the recombination rate of electrons, U_P_ is the recombination rate of holes, and J_n_ and J_p_ are, respectively, the current densities of electrons and holes. Also, G_p_ and Gn are the total electron and hole generation rates, for which we assumed that G_n_ = G_p_ = G_opt_. By optimizing one of the two parameters of J_sc_ and V_oc_, PCE can be increased. J_sc_ is significantly improved by changing the absorption of the active layer. This improvement in absorption is directly related to Eq. (3). For this reason, by increasing the thickness of the active layer, the absorption of this layer also increases. However, increasing the thickness leads to an increase in the recombination rate of the carriers and affects the carrier transfer, which reduces the V_oc_. In order for PCE to increase, both the optical model and the electrical model of the device must be in proper equilibrium. Therefore, by using a suitable light management plan, the absorption rate can be increased without changing the thickness of the active layer, followed by the Gopt. Also, the use of a complementary material that can be very effective in the G_opt_ and lead to the production of more J_sc_^84–86^.

### Simulation details

The proposed nanostructure design is based on electronic and photonic considerations. The presented nanostructures were assessed for two photonic architectural considerations: first, optical trapping is accomplished by orthogonalizing carrier transport and photon propagation. If incident photon dispersion generally takes place above the active region, where nanostructure would lead the CH_3_NH_3_PbI_3_/HTM interface to be in the normal direction above the active layer, then this might be feasible. As a result, the movement of charge carriers across interfaces must be in opposition to the movement of photons. The distance between nanostructures in a repeating pattern affects LT as well. In order to increase absorption, we firmly pack the nanostructures into grids with rising aspect ratios. Enhancing antireflection is the second photonic factor to take into account. It has been confirmed that the graded index of optical impedance matching is a powerful antireflection strategy^87^. There are two geometric prerequisites for an anti-reflection coating to be effective. To properly average the refractive index, the period of the nanostructures must be significantly smaller than the wavelength of the incident light. Second, for smooth transitions, the nanostructure height needs to be sufficiently large. The absorption should get better if each of these conditions are met throughout a wide spectrum of wavelengths. Contrarily, two distinct electronic uses while creating nanostructures are: maximize the number of electron–hole pairs that are produced first. Nanostructures with dimensions shorter than the diffusion length can be used to accomplish this. The electron diffusion length of CH_3_NH_3_PbI_3_ is greater than 1 μm, which is equivalent to an intensity of light of more than four orders of magnitude. Increasing the internal electric field to increase the charge carrier collecting rate is the second electronic aspect in our method. In order to improve light absorption, the thickness of the nanostructures should be greater than the thickness of the space-charge area^88^. According to the optical and electrical models, we can get the J_sc_ and V_oc_ for the proposed PSC. In the optical model, first the Maxwell equations are solved for the whole device, then using Eq. (3), the carrier generation rates are obtained. By placing the values obtained from Eq. (3) at each wavelength with a resolution of 20 nm in the electric model, J_sc_ and V_oc_ are obtained. The basic (planar) structure (Fig. 1a) consists of layers of ITO (as front contact), TiO_2_ (as ETL), CuSCN (as HTL), CH_3_NH_3_PbI_3_ (as an adsorbent layer), and Au metal as the back contact. In order to subtract the volume of computation, we considered a unit cell of the whole structure, and periodic boundary conditions (PBC) were used as the boundary conditions to approximate the whole structure. Two-dimensional (2D) simulation is performed in two electric-optical models with an input power of standard AM1.5G spectrum as a sunlight simulator. The wavelength range for the structure of PSC with active layer CH_3_NH_3_PbI_3_ is from 300 to 800 nm and for PSC with layer CH_3_NH_3_SnI_3_ from 300 to 1000 nm with steps of 20 nm. In the optical model, all the layers are placed and the complex refractive indices of ITO, CuSCN, CH_3_NH_3_SnI_3_, CH_3_NH_3_PbI_3_ and TiO_2_ are taken from the previous studies^89–96^. SRH recombination is used to simulate the recombination of carriers in the electric model. Also, the ITO and metal contact are considered to be ohmic and Shockley, respectively. For surface recombination, the velocities of electrons and holes are set to Sn = Sp = 1 × 10^7^ cm/s. The parameters of the selected layers in the electrical model are extracted from^97–111^ references. Also, the electrical parameters are summarized in Table 1.

**Table 1:** Electrical parameters required for PSC.

Parameter	TiO_2_	CH_3_NH_3_PbI_3_	CH_3_NH_3_SnI_3_	CuSCN
Ꜫ_r_	9	6.5	8.2	10
N_C_ (cm^−3^)	1 × 10^19^	1.66 × 10^19^	1 × 10^18^	1.79 × 10^19^
N_V_ (cm^−3^)	1 × 10^19^	5.41 × 10^19^	1 × 10^18^	2.51 × 10^19^
µ_n_/µ_p_ (cm^2^/V)	20/10	20/50	50/50	25/25
χ (eV)	4	3.93	4.17	1.9
E_g_ (eV)	3.2	1.55	1.3	3.4
N_A_ (cm^−3^)	–	5 × 10^13^	1 × 10^16^	5 × 10^18^
N_D_ (cm^−3^)	5 × 10^18^	–	–	–
τ_n_/τ_p_ (ns)	5/2	8/8	25/25	5/5

### Analyzing the suggested PSC's structural organization

Wet anisotropic etching can etch silicon to the desired structure using a bulk micro-machining process. Depending on the silicon's crystallographic orientation and the type of etchant used, one can create three-dimensional shapes using this technique, which has the advantages of being inexpensive, fast, and highly selective. Since the silicon crystal's (100) plane etching occurs more quickly and at a higher rate than the (111) plane, angular shapes can be created by etching the silicon crystal (100). The etching is much slower in the (111) plane, which results in this angle, which is 54.7 degrees, between the (100) and (111) planes. Thus, the proposed structure can be created with a smooth surface by using the proper orientation of crystalline silicon, a suitable etchant and etching time, and a suitable temperature.

V-shaped and pyramidal structures have traditionally been created on silicon surfaces using photolithography and wet etching methods. In this procedure, a silicon (100) wafer is first covered with a Si_3_N_4_ sheet, typically by evaporation, and a photoresist (PR) film is subsequently deposited by spin coating. Once employing photolithography to create the required pattern on the PR, the PR photo-mask is entirely removed once the Si_3_N_4_ etching barrier has been selectively etched using the buffered hydrofluoric (HF) acid etchant. The ideal V-shaped structure with smooth surfaces and tips is created by submerging the wafer in the KOH solution at temperatures between 80 and 85 °C in the procedure that follows. In the final, the sample is submerged in diluted hydrogen fluoride to thoroughly eliminate the Si_3_N_4_^112–114^. Maintaining the quality of the deposited films on the silicon substrate is essential since the suggested PSC must be constructed from the bottom layer up to create the necessary light trapping structure. We can leverage fabrication techniques used to create inverted perovskite solar cells (IPSC) to obtain the desired high efficiency. This is due to studies showing that by employing suitable deposition techniques, high efficiency may be achieved in IPSCs, in which the coating of the films is done from the bottom layer. In this manner, a high-quality perovskite film on CuSCN layer with low surface roughness and modest series resistance can be created in a single phase of quick deposition and crystallization. By adjusting the electrodeposition time of the CuSCN film, the thickness of the film can be changed in this procedure. In addition, by using the Brookite-phase TiO_2_ top buffer on perovskite film, high efficiency and long lifetime IPSC can be obtained in addition to enhanced carrier extraction and transport^115–118^.

A conformal perovskite film and other PSC components can be made using a variety of techniques, including evaporation, vacuum deposition, atomic layer deposition, sputtering, etc. on a textured substrate. In this sense, perovskite can be deposited on a subordinate textured silicon wafer using a two-step hybrid process that combines co evaporation and spin coating. Studies have also demonstrated that a high-roughness surface can be used to produce homogenous perovskite films using the sputter-based method. Additionally, if a dry two-step deposition procedure is utilized, the precursor composition, temperature, and conversion time can all be varied to alter the perovskite film's quality^119–124^.

## Results

**Figure 2:**
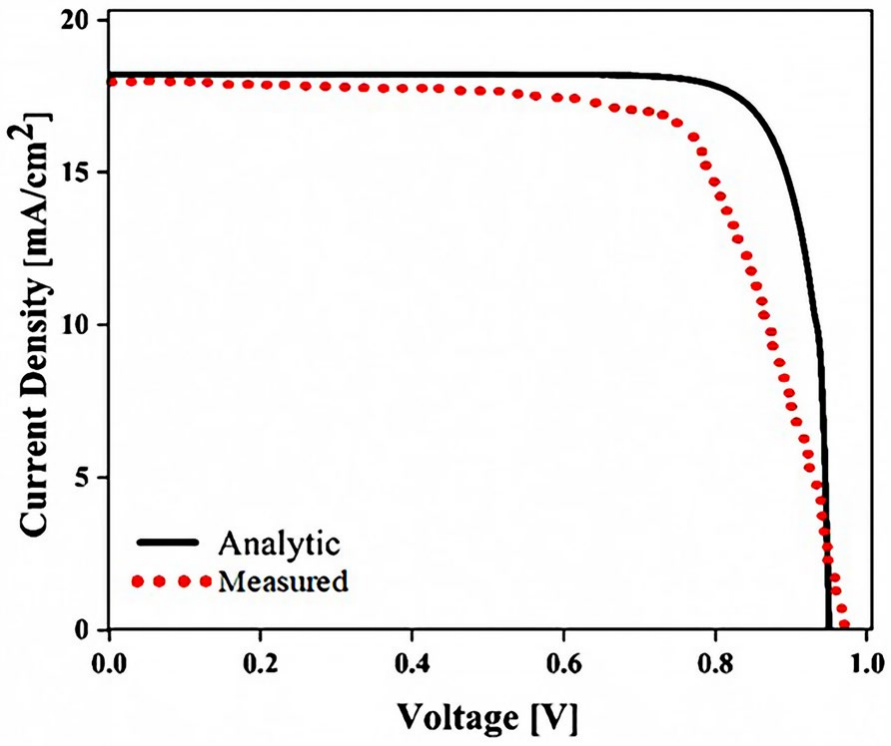
J-V specifications of the same PSC structure in both measurement and analytic modes.

**Figure 3:**
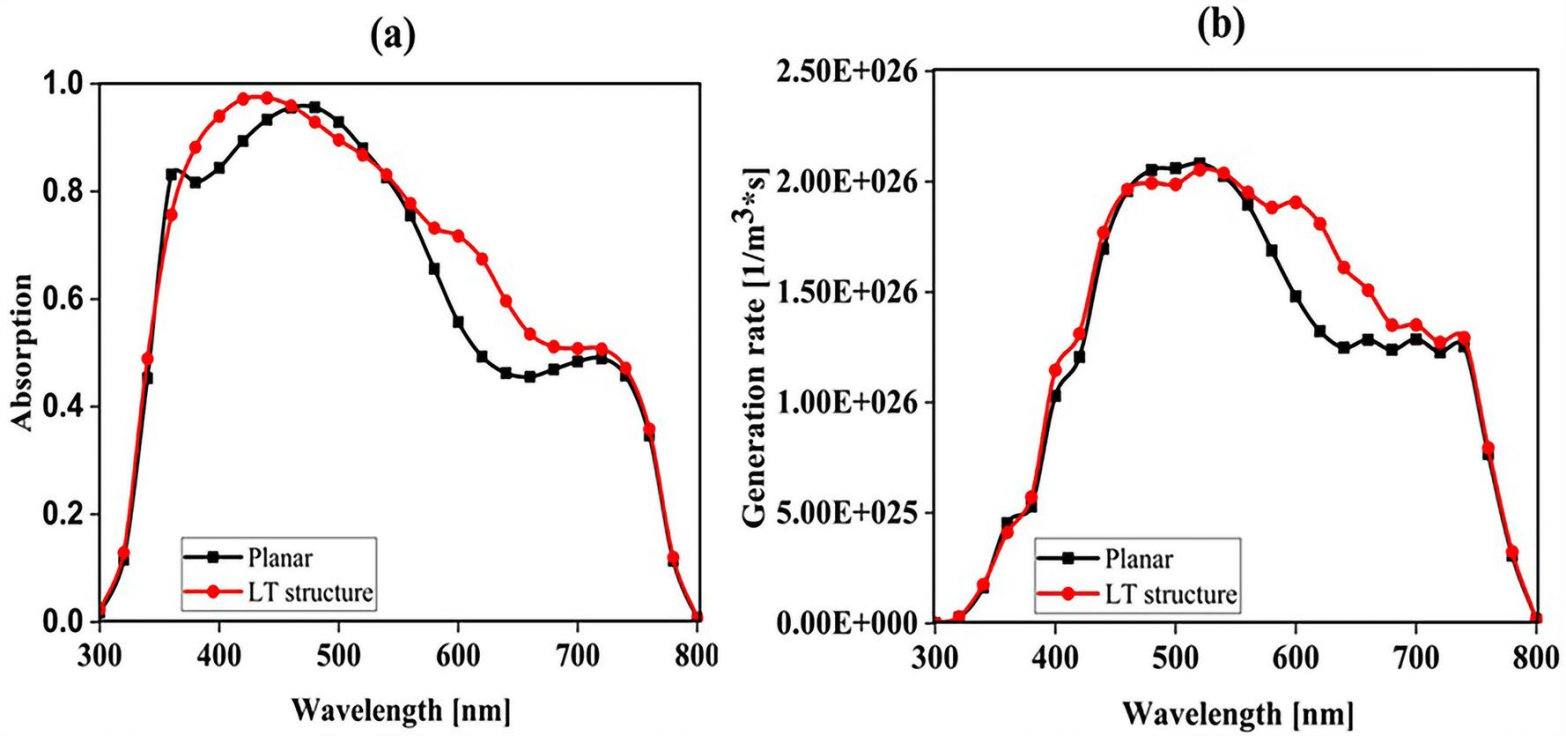
Diagram of (**a**) absorption and (**b**) carrier generation rate (G_opt_) in the active layer (CH_3_NH_3_PbI_3_) for both planar and LT structures in terms of wavelength.

**Figure 4:**
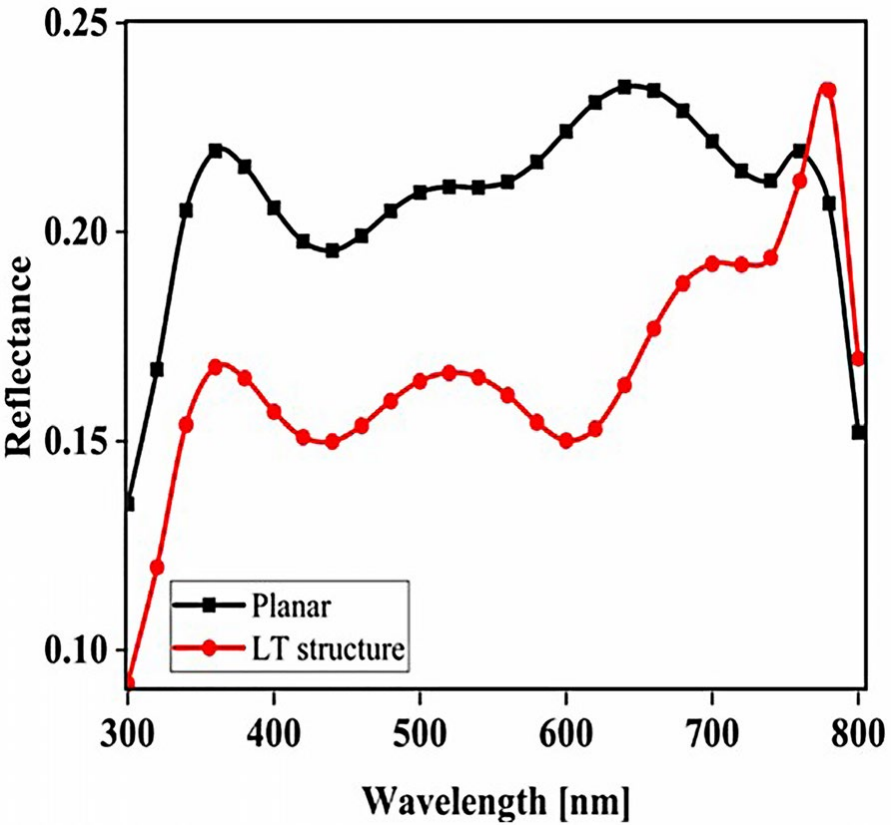
Total reflection from the surface of CH_3_NH_3_PbI_3_ in both planar and LT structures.

**Figure 5:**
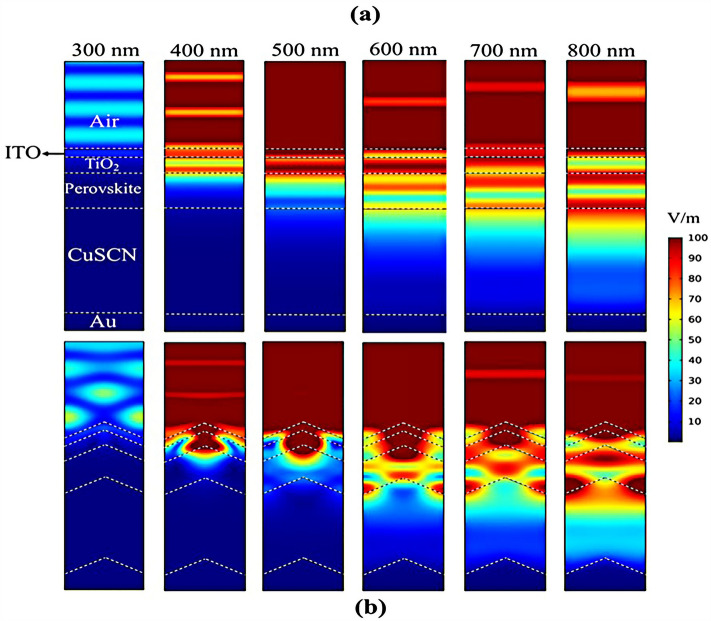
Electric field profiles in all PSC layers in (**a**) planar structure and (**b**) LT structure.

**Figure 6:**
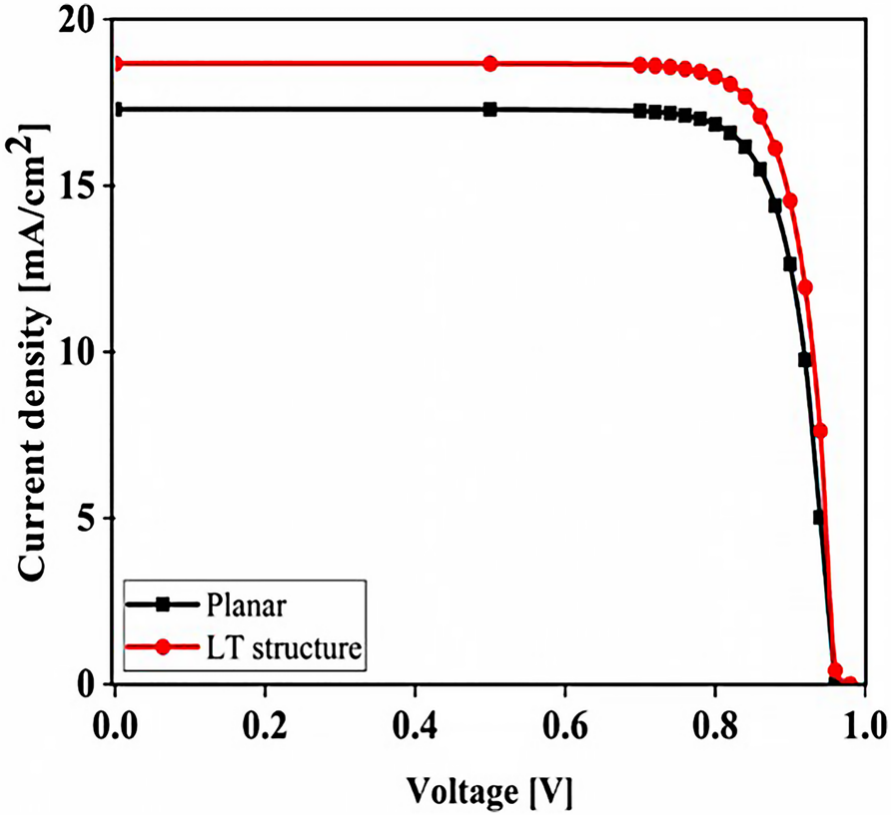
Comparison of J_sc_-V_oc_ characteristics for both planar and LT structures in PSC.

The planar configuration seen in Fig. 1a was the first structure we simulated. The five layers of the structure are depicted in the diagram. To control the efficacy of this method, planar structures were simulated using the previously stated computation process, with ITO, TiO_2_, perovskite, CuSCN, and Au having thicknesses of 50, 90, 200, 600, and 100 nm, respectively. In order to get the right findings, it was therefore regarded as the fundamental hardware for subsequent simulations. To increase the output of this kind of PSCs, the proposed structure (planar structure) was applied. The simulation was run using the procedure depicted in Fig. 1a. The graphic also displays the size and composition of the materials. The values for this structure short circuit current (J_sc_), open circuit voltage (V_oc_), power conversion efficiency (PCE), and fill factor (FF) were, respectively, 17.29 mA/cm^2^, 0.94 V, 13.40%, and 82.50. The findings of our simulation of the structure as it is described in^125^, indicated that the PSC variables closely matched the experimental data. Figure 2 displays these findings. It should be emphasized that there were variances from experimental findings in various electrical parameters, such as V_oc_. This discrepancy might exist because the simulation model excluded variables that might have increased resistance between the layers, such as recombination between layers, temperature problems, or mismatch between each layer caused by the manufacturing process. Nonetheless, efforts were made to ensure that the models and tests were as closely matched as feasible. Many organic and inorganic compounds are examined as HTL in^126^. The researched HTL materials are: Spiro-OMeTAD [(2,2′,7,7′-tetrakis-(N, Ndi-p-methoxyphenyl-amine) 9,9′spirobifluorene)], P_3_HT [poly (3-hexylthiophène-2,5-diyl)], CuI [Copper(I) iodide] CuSCN [Copper(I) thiocyanate], and NiO [Nickel (II) Oxide]. P_3_HT, Spiro-OMeTAD, NiO, CuSCN and CuI each have input variables that were taken from^126–130^, Since the difference between the HOMO levels of P_3_HT and MAPbI_3_ reached 0.5 eV, the solar cell utilizing P_3_HT as the HTL generates the lowest efficiency of power conversion, according to^126^. Yet, even though Spiro-OMeTAD shows the finest band alignment, CuSCN showed the best efficiency. This is because the mobility of the holes in Spiro-OMeTAD is substantially lower than that of the holes in CuSCN. When CuSCN is utilized as the HTL, the solar cell displays a high fill factor of FF 83.70% and a conversion efficiency of PCE 23.30%. In this section, we discuss the simulation results from Fig. 1a,b. The most important factors in a solar cell are the absorption diagram and the carrier generation rate. Because by using these two factors, the optical part of the solar cell can be examined, these two factors must be optimized to maximize the efficiency. Therefore, the diagram of absorption and carrier generation rate for both planar and LT structures is shown in Fig. 3. According to Fig. 3a, when we changed the planar structure to LT, the absorption of the active layer (CH_3_NH_3_PbI_3_) generally increased in the two regions. (The first region is between wavelengths of 400–500 nm and the second region is between wavelengths of 550–700 nm). The reason for this increase in absorption can be attributed to the LT in the structure. On the other hand, due to the morphology of the structure used, the incoming light enters the active layer more easily and it is harder to get out of it. In other words, the LT structure in the PSC creates a grating and reduces the amount of light reflection (explained earlier in the amount of light reflection). For this reason, light absorption in the CH_3_NH_3_PbI_3_ is generally increased. After obtaining the electric field in all the layers, we now calculate the carrier generation rate for all wavelengths using Eq. (3). Figure 3b shows the carrier generation rate for the planar structure and LT in the CH_3_NH_3_PbI_3_. The carrier generation rate spectrum behaves almost identically to the absorption spectrum, and at wavelengths of 550 to 700 nm, the carrier generation rate increases relative to the planar structure due to LT in the active layer. As mentioned, we calculated the amount of reflection from the surface of the CH_3_NH_3_PbI_3_ in both planar and LT structures, which is shown in Fig. 4. It should be noted that the amount of reflection is only from the surface of the active layer and not the total reflection. As shown in Fig. 4, when the LT nanostructure was used, the reflectance was lower than in the planar state. This means that light easily enters the active layer and is confinement inside the active layer. Also, because light is difficult to exit, the volume of light entering the active layer increases as the wavelength increases. In order to better explain how light functions in the LT structure, the electric field profile for a specific range of wavelengths (300–800 nm) are shown in Fig. 5. Figure 5a shows how the electric field is distributed for a planar structure. At a wavelength of 400 nm, some light enters the active layer, while in Fig. 5b, at the same wavelength, due to the LT structure, the volume of light entering is increased. And this behavior is quite evident at all wavelengths. Another advantage of the LT structure used is the reduced light reflection as well as light confinement on both sides of the LT structure (in Fig. 5b, at 800 nm, this phenomenon is quite clear). All these factors minimize light loss and increase cell performance in the optical model. Using the electric model, we calculated the value of V_oc_ and, through Eq. (4), we obtained the J_sc_ diagrams in terms of V_oc_ for both planar and LT structures, and it is shown in Fig. 6. As expected, because G_opt_ was larger in the LT structure than in the planar structure, the J_sc_ rate also increased. For a more detailed analysis, the other electrical parameters of these two structures are listed in Table 2. According to this table, the J_sc_ value for the planar structure is 17.29 (mA/cm^2^), while this parameter was 18.67 (mA/cm^2^) for the LT structure. The PCE of PSC with a LT structure has increased by 10%. Influence of adding SiO_2_ layer as anti-reflectance on LT structure.

**Table 2:** Electrical parameters for PSC with four structures.

Structure	J_sc_ (mA/cm^2^)	V_oc_ (V)	FF (%)	PCE (%)
Planar structure	17.29	0.94	82.50	13.40
LT structure	18.67	0.94	82.51	14.50
LT structure with SiO_2_	19.52	0.94	82.51	14.93
LT structure with SiO_2_ and CH_3_NH_3_SnI_3_	21.97	0.96	83.33	17.57

### Influence of adding SiO_2_ layer as anti-reflectance on LT structure

**Figure 7:**
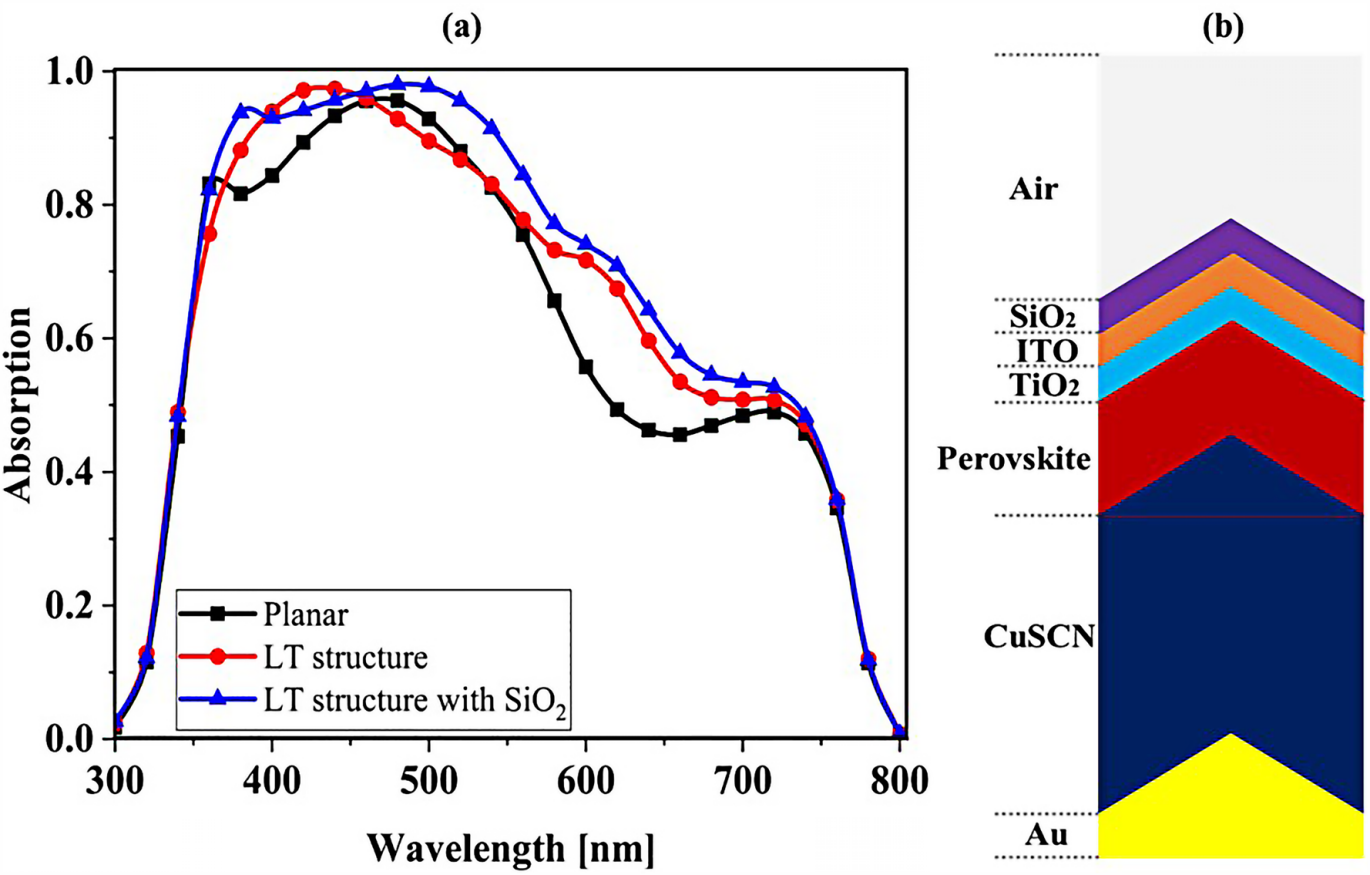
(**a**) Changes of active layer absorption by adding SiO_2_ layer as anti-reflectance in LT structure and (**b**) Schematic of LT structure in the presence of SiO_2_ layer.

**Figure 8:**
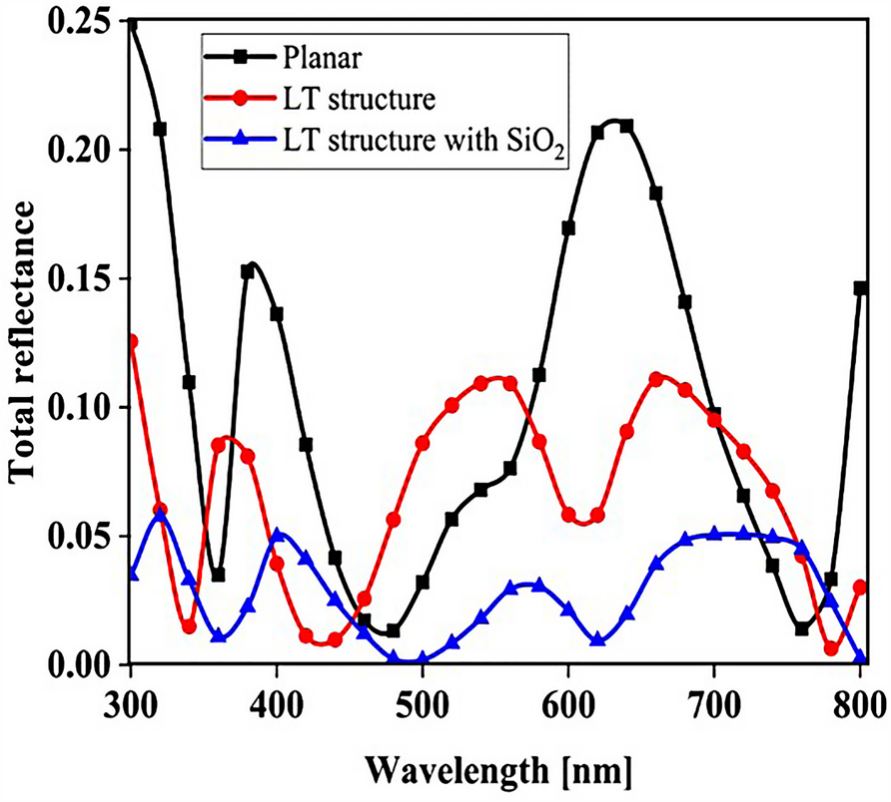
Total reflectance in terms of wavelength for planar and LT structures and SiO_2_ layer in PSC.

**Figure 9:**
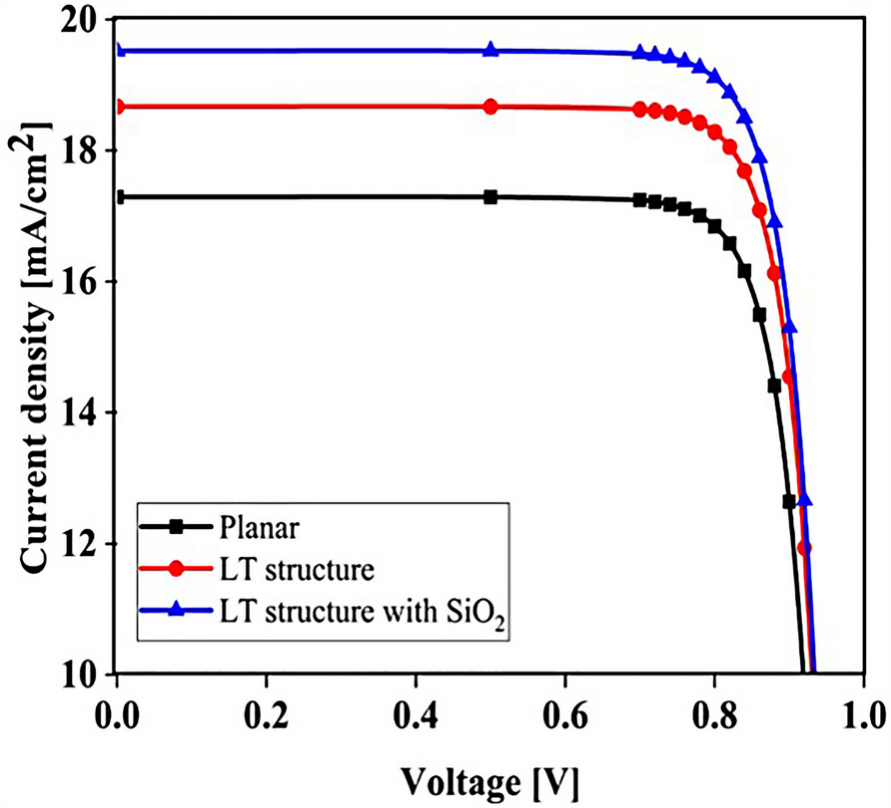
PSC J_sc_-V_oc_ curve in the presence of SiO_2_ layer in LT structure.

To be able to reduce the amount of light reflection initially entering the structure, we used an anti-reflective SiO_2_ layer with an LT structure on top of the ITO layer (Fig. 7b shows a schematic of the structure). To investigate whether the addition of the SiO_2_ layer affects the absorption, we obtained the absorption diagram in the presence of the SiO_2_ layer (Fig. 7a). As it turns out, the amount of absorption is higher when the SiO_2_ layer is in the LT structure than in the other two states. The reason for this can be seen in the amount of light entering the active layer. In fact, the amount of reflection is reduced, and as a result, a volume of light is able to reach the active layer. To better understand whether the reflection rate has decreased, we obtained the total reflectance for the three structures, including planar, LT, and SiO_2_. According to Fig. 8, the reflection rate decreased when we used the LT structure. In the next step, by adding a SiO_2_ layer to the same structure, the reflection rate was significantly reduced. This indicates that more light has entered the proposed PSC and leads to increased absorption and subsequent G_opt_. In this section, we show the J_sc_-V_oc_ curve for the proposed PSC in Fig. 9. When the SiO_2_ layer was placed, according to the description given, the current also increased to 19.52 (mA/cm^2^) and the PCE in the presence of the SiO_2_ layer increased to 14.93%.

### Influence of adding CH_3_NH_3_SnI_3_ layer to increase performance and improve carrier generation rate

**Figure 10:**
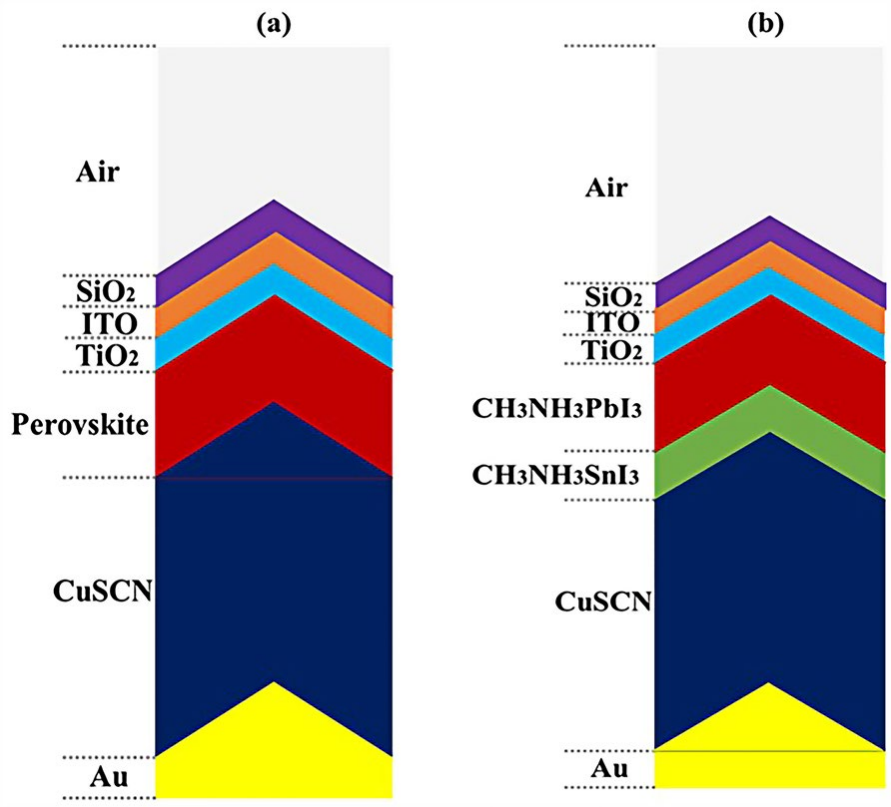
Schematic of LT structure for PSC (**a**) without CH_3_NH_3_PbI_3_ layer and (**b**) with CH_3_NH_3_PbI_3_ layer.

**Figure 11:**
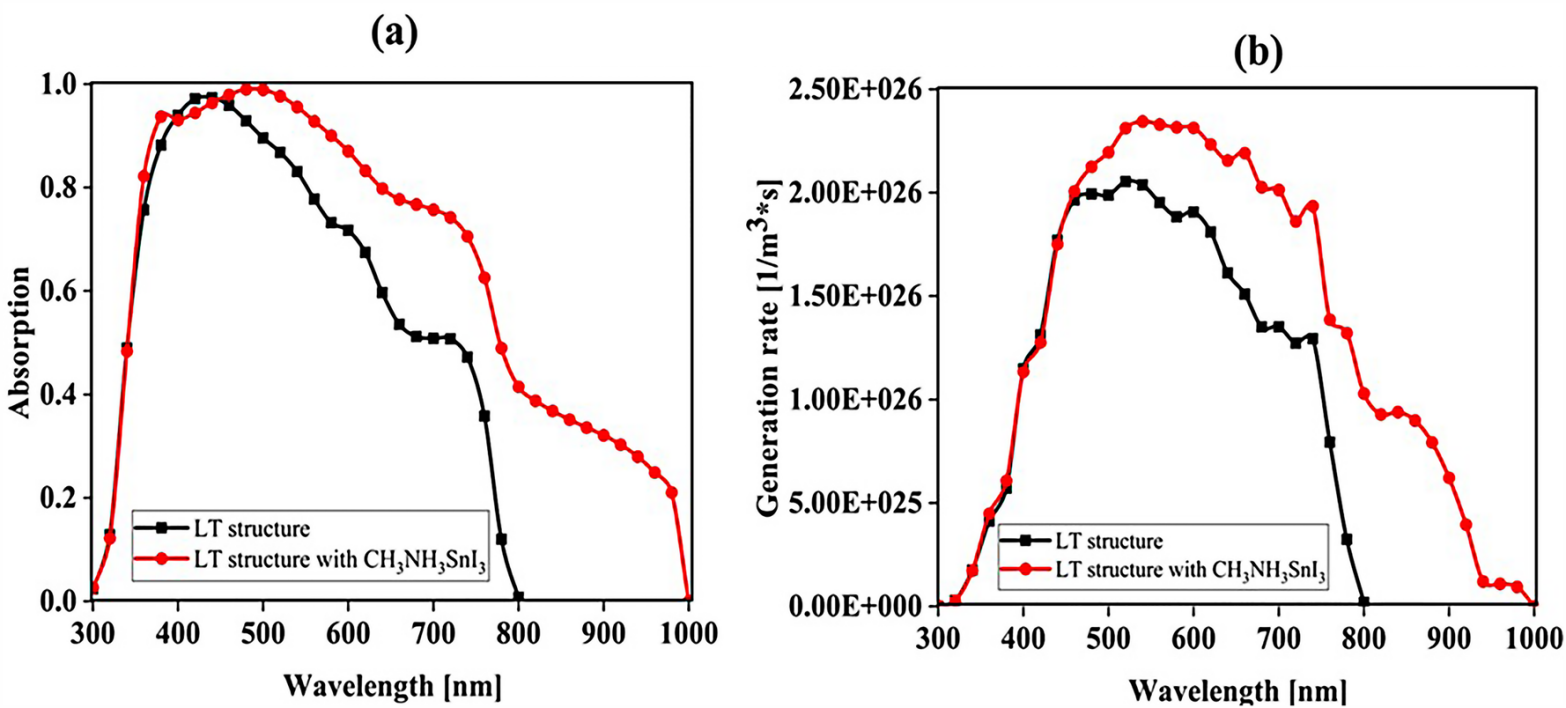
Diagram of (**a**) absorption and (**b**) carrier generation rate (G_opt_) in the active layer in terms of wavelength for the LT structures with and without CH_3_NH_3_SnI_3_ layer.

**Figure 12:**
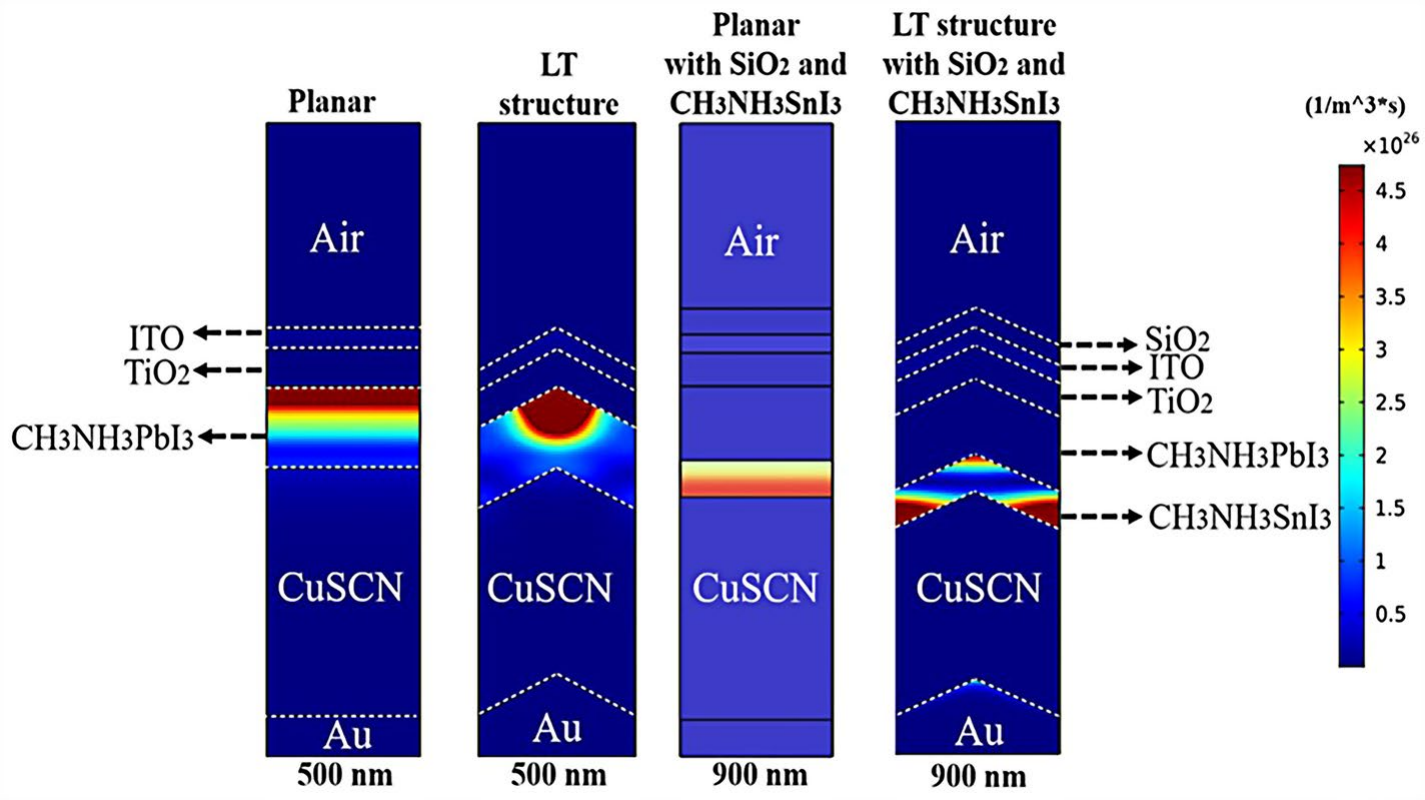
G_opt_-profiles in three different structures including planar and LT for PSC and the impact of CH_3_NH_3_SnI_3_ layer on G_opt_.

**Figure 13:**
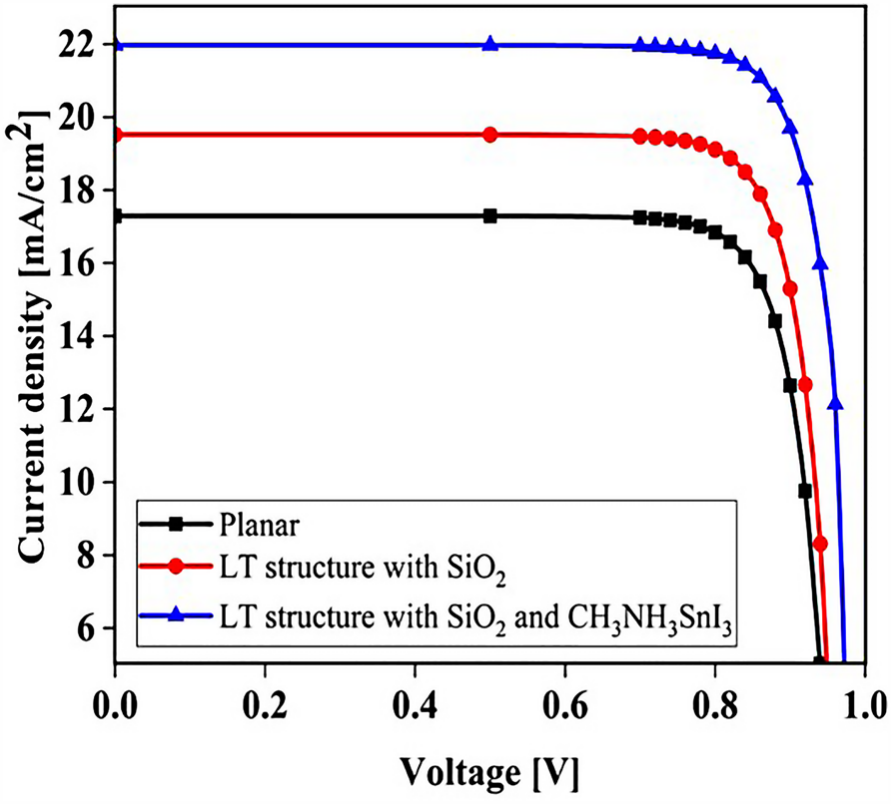
Comparison of J_sc_-V_oc_ characteristic of PSC in three modes of planar structure (black line), LT structure with SiO_2_ (red line) and LT structure with SiO_2_ and CH_3_NH_3_SnI_3_ (blue line).

**Figure 14:**
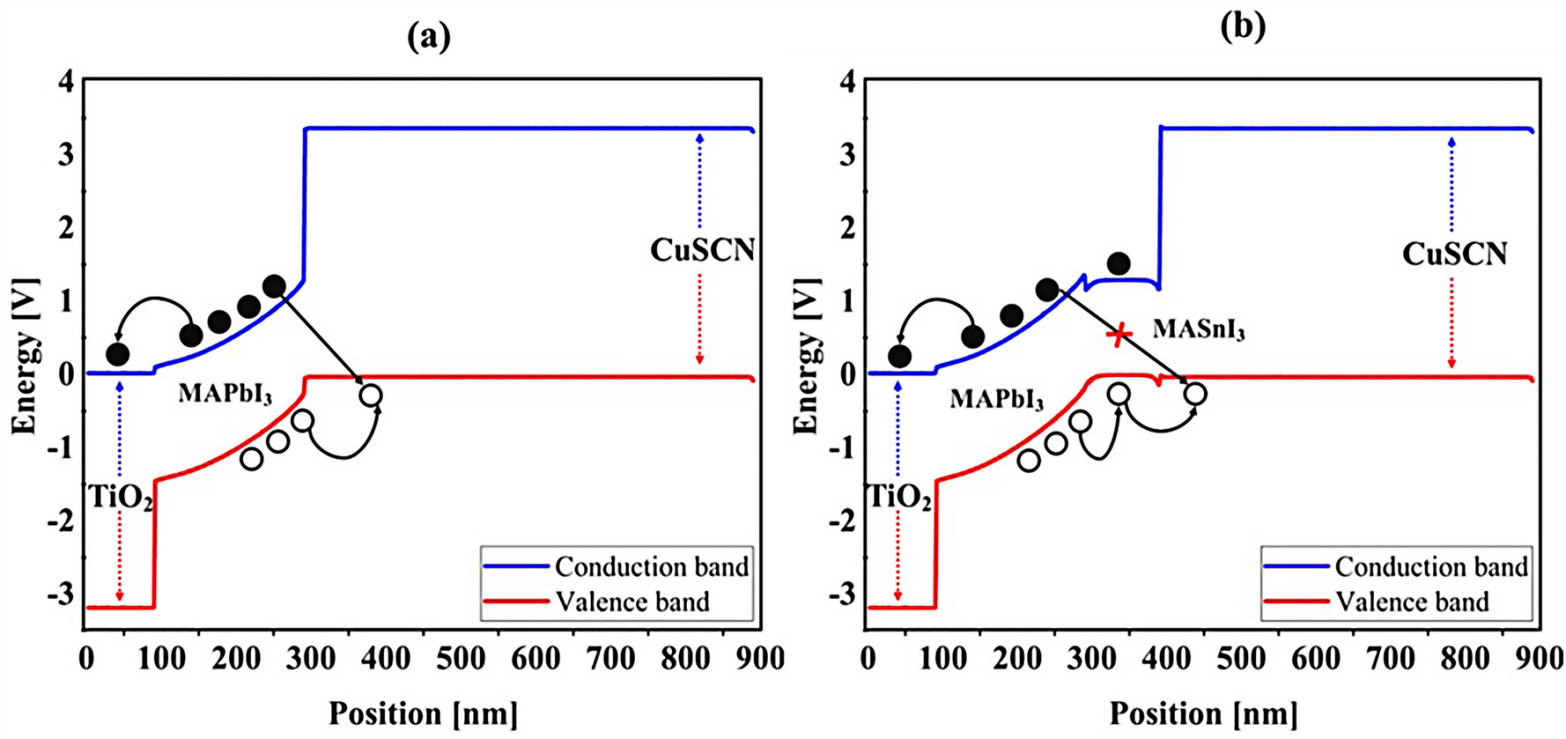
Band diagrams of the carrier transfer and recombination in the PSC for (**a**) without MASnI_3_, and (**b**) with MASnI_3_.

**Figure 15:**
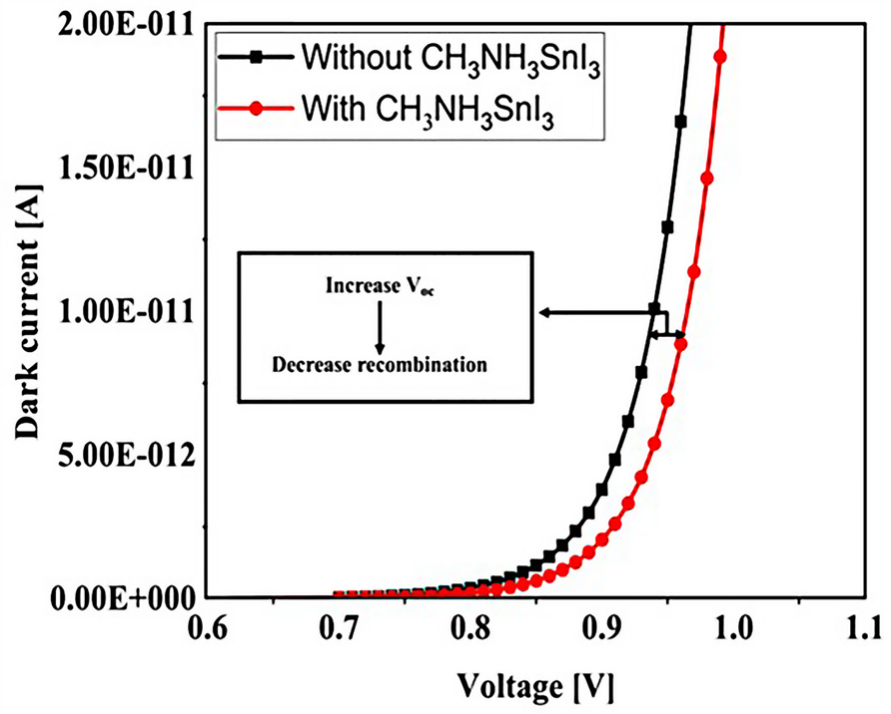
The dark current versus V_oc_; for with and without MASnI_3_.

An important strategy to improve PSC performance is to limit the absorption spectrum of the solar cell to the near-infrared spectrum. Because of the perovskite CH_3_NH_3_PbI_3_ band gap of about 1.55 eV, the amount of light absorption by this type of solar cell for wavelengths greater than 800 nm is extremely low. Ideally, solar cells should be able to absorb the visible part and part of the near-infrared spectrum, which will improve solar cell efficiency due to the absorption of more parts of the solar spectrum. The highest theoretical efficiency for a single-junction solar cell with a band gap of about 1.1 eV, which is capable of absorbing photons with energy higher than this value (λ 1227 [nm]), is about 30%. As stated in section "Introduction", perovskite refers to any material with the structure ABX_3_, and different materials have this structure. For example, the CH_3_NH_3_SnI_3_ (MASnI_3_) material is a perovskite material. In conventional PSC, because the band gap of CH_3_NH_3_PbI_3_ is about 1.55 eV, only photons with energy levels below this value (photons with a wavelength of less than 850 nm) are absorbed into the structure. According to this point, if a material with a band gap of less than 1.55 eV is added to the structure, it is possible to absorb photons with less energy in the solar cell. A wider range of the sun’s spectrum will be absorbed in the solar cell. So, to achieve this goal, we placed another layer of MASnI3, which has a crystal lattice structure similar to CH_3_NH_3_PbI_3_ but has a band gap of about 1.3 eV, between HTL and layer CH_3_NH_3_PbI_3_. Figure 10 shows schematic of LT structure for PSC (Fig. 10a) without CH_3_NH_3_SnI_3_ layer and (Fig. 10b) with CH_3_NH_3_SnI_3_ layer. As shown in Fig. 10b by adding this layer to the structure, Therefore, it will increase the J_sc_ (J_sc_ = J_photon_). In many reports, the electron acceptor density amount for the MASnI_3_ layer is selected above 1 × 10^16^ cm^−3^. Also, in works^75,131–133^, the MASnI_3_ layer is used as a complementary layer next to the MAPbI_3_ layer. It is also stated in the report of^133^ work that these two active material (MAPbI_3_ and MASnI_3_) can improve the overall performance of the device together. Now, in order to investigate the effect of this layer on the absorption and G_opt_, we must repeat the steps mentioned in section "Methods" in order to simulate this type of solar cell. As shown in Fig. 10b, a 100 nm thick layer of CH_3_NH_3_SnI_3_ (MASnI_3_) material is placed between the CuSCN and CH_3_NH_3_PbI_3_ layers. After simulating how the electromagnetic wave propagates in this structure, absorption and G_opt_ are obtained and are shown in Fig. 11. As shown in Fig. 11a, the absorption increased between the wavelengths of 300 and 800 nm, and this can be attributed to the positive effect of the absorption of the CH_3_NH_3_SnI_3_ layer on the total absorption. However, from the wavelength of 800 to 1000 nm, a part has been added to the absorption diagram of the active layer, which is due to the presence of the CH_3_NH_3_SnI_3_ layer. In this regard, G_opt_ in Fig. 11b behaves almost similarly to the absorption spectrum, and at wavelengths after 800 nm, some G_opt_ is added to the structure, which causes the J_sc_ to increase. Figure 12 shows an example of the values obtained for G_opt_ at several wavelengths. According to Fig. 12, by comparing the G_opt_-profile for the two planar and LT structures, it is clear that because of the presence of the LT structure, the G_opt_ due to LT has increased, which increases the J_sc_. It is worth noting that by comparing the G_opt_ obtained for 900 nm in PSC including the CH_3_NH_3_SnI_3_ layer with the LT structure without the CH_3_NH_3_SnI_3_ layer, it is clear that at this wavelength in the CH_3_NH_3_SnI_3_ layer, light absorption occurs. Therefore, by adding this layer to the LT structure in PSC, it achieved the desired goal, which is to increase the range of light absorption in PSC. In this case, for the solar cell containing the CH_3_NH_3_SnI_3_ layer, the current density curve in terms of voltage was obtained according to Fig. 13. Figure 13 shows the J_sc_-V_oc_ diagram of PSC in three modes of planar structure: LT structure with SiO_2_, LT structure with SiO_2_ and CH_3_NH_3_SnI_3_, respectively. As it is known, the use of bilayer as an anti-reflective layer and light absorber (SiO_2_ and CH_3_NH_3_SnI_3_) in the form of an LT structure has improved the J_sc_. Table 2 summarizes the photovoltaic parameters obtained for all three types of PSC. According to this table, the best J_sc_ and PCE were related to LT structure with SiO_2_ and CH_3_NH_3_SnI_3_ with values of 21.97 mA/cm^2^ and 17.57%, respectively. But for further explanation and how carriers are transported in the presence of MASnI_3_ layer, we have shown the energy balance diagram as well as the dark current diagram which shows the recombination rate. Figure 14 shows the band diagram of the carrier transfer and recombinant in the PSC for the without and with MASnI3. The holes created in the perovskite can transmit straight to the HTL due to the interaction between the HTL and perovskite layers (Fig. 14a). The presence of MASnI_3_ layer makes the recombination in the system less. According to Fig. 14a, due to the low bandgap of the MAPbI_3_ layer, it increases the possibility of carrier recombination in the MASnI_3_ and HTL layers. But the MASnI_3_ layer acts like an interlayer and minimizes the possibility of carrier recombination between the MAPbI_3_ and HTL layers. As can be seen in Fig. 14b, after electron–hole pairing in MAPbI_3_, holes are able to transfer despite facing an energy barrier of less than 0.1 eV. As a result, holes can migrate through MASnI_3_, a material with high hole mobility and high life duration, more quickly and toward the HTL. These observations lead us to the conclusion that the MASnI_3_ lowers carrier (hole) recombination and charge accumulation. Consequently, the carrier recombination in PSC is reduced as a result of altering the layer structure and introducing the MASnI_3_ layer, which ultimately enhances cell performance. The dark current parameters have been retrieved and are depicted in Fig. 15 to support the improvement in structure caused by recombination. This graph makes it obvious that the MASnI_3_ causes a rise in the amount of dark current. This increase in dark current with the addition of MASnI_3_ suggests that the produced carriers are transmitted to the primary HTL (CuSCN) prior to recombination, which improves V_oc_.

### Influence of SiO_2_ layer thickness to optimize the absorption parameter

**Figure 16:**
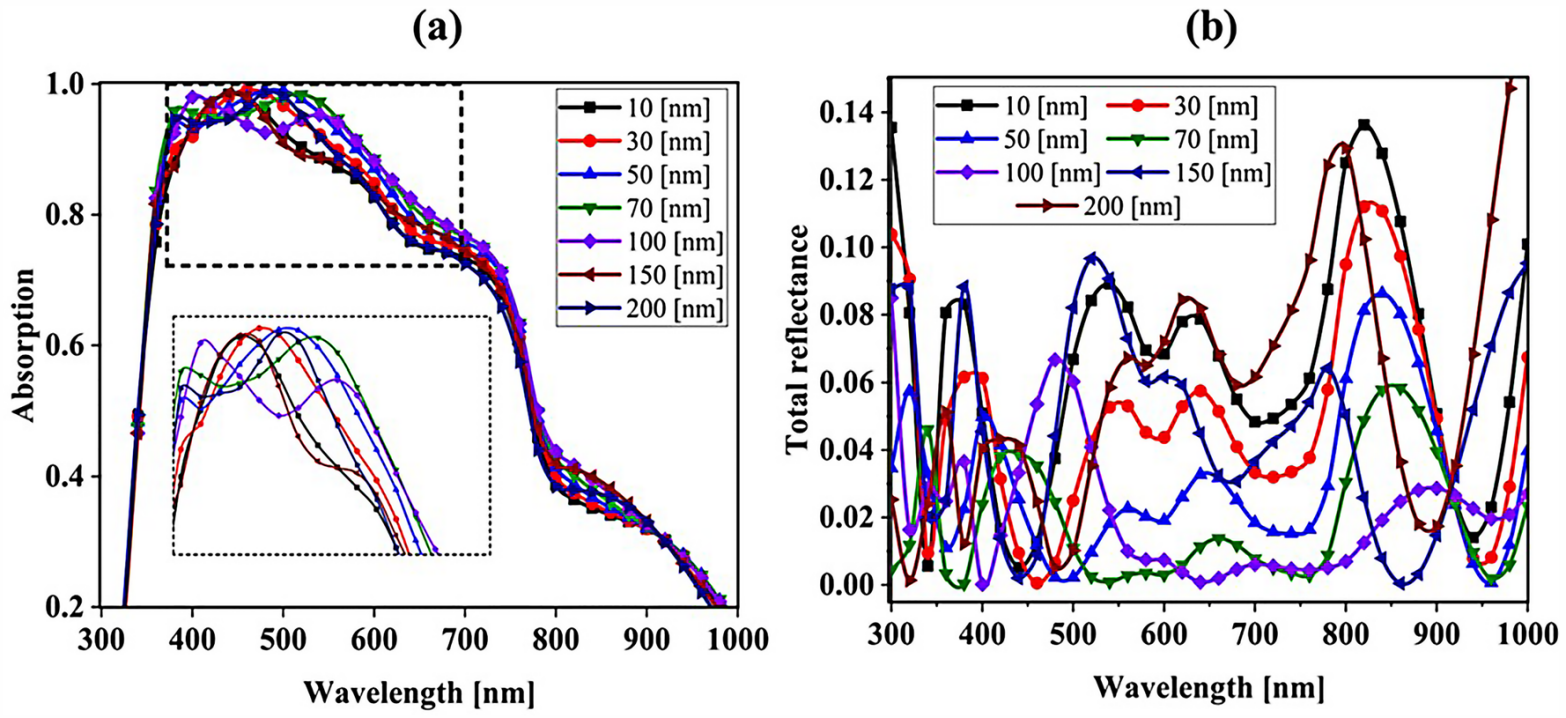
Influence of SiO_2_ layer thickness on CH_3_NH_3_PbI_3_ and CH_3_NH_3_SnI_3:_ (**a**) absorption and **(b**) total reflection.

**Figure 17:**
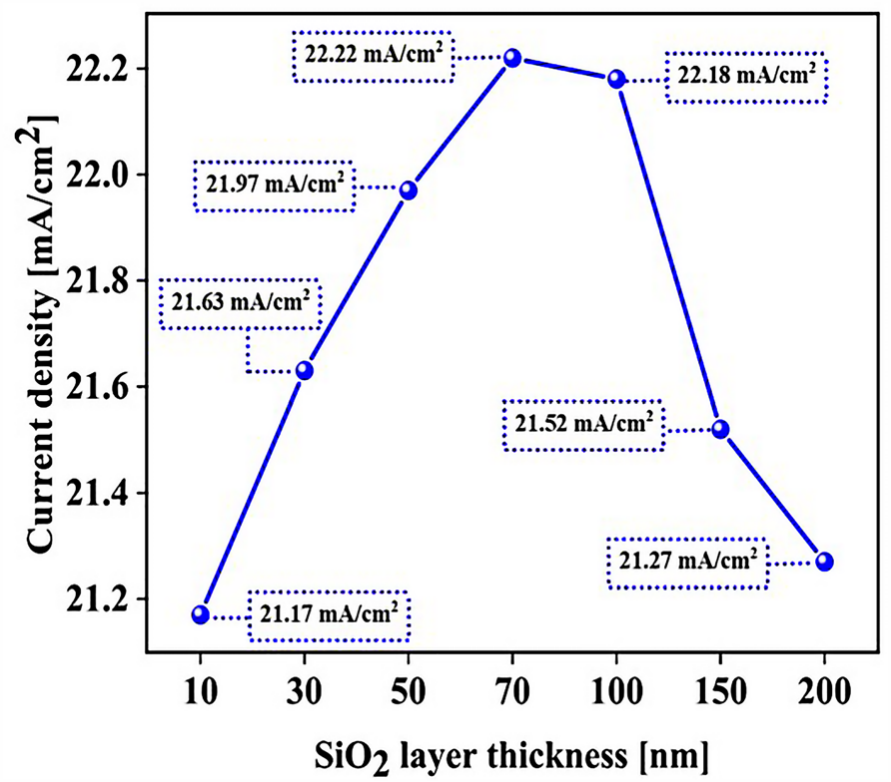
Current density for different thicknesses of the SiO_2_ layer.

To achieve greater efficiency, we examined the thickness of the SiO_2_ layer at the end. For this purpose, we obtained the absorption and total reflection for different thicknesses of the SiO_2_ layer. Figure 16 shows the influence of the SiO_2_ layer thickness on the CH_3_NH_3_PbI_3_ and CH_3_NH_3_SnI_3_ (Fig. 16a) absorption and (Fig. 16b) total reflection. According to Fig. 13a, the absorption changes in the range of 400–700 nm are very noticeable. By changing the thickness of the SiO_2_ layer from 10 to 70 nm, the amount of absorption increases. At low thicknesses (10–30 nm), the anti-reflective effect of SiO_2_ is low, so the absorption of the perovskite layer does not change much. At higher thicknesses (50–70 nm), the SiO_2_ layer was effective in absorption. This issue is quite evident in Fig. 13b because it is directly related to the total reflection. To see if increasing the thickness helps to improve the absorption, we increased the thickness of the SiO_2_ layer to 200 nm and found that at thicknesses above 70 nm, this upward trend in absorption does not occur. According to Fig. 16b, the reflection of the whole system increased with increasing thickness up to 200 nm, while we expected the reflection rate to decrease. By increasing the thickness of the SiO_2_ layer at high thicknesses (200 nm), this layer no longer has an anti-reflective role and prevents light from entering the system. Figure 17 shows the current density (J_sc_) rate based on the thickness of the SiO_2_ layer. The best J_sc_ was 70 nm thick, i.e., 22.22 mA/cm^2^, and also with other parameters of the solar cell were: V_oc_, FF, and PCE, were 0.96 V, 83.33, and 17.77%, respectively.

### Influence of MASnI_3_ layer thickness to optimize the absorption parameter

**Figure 18:**
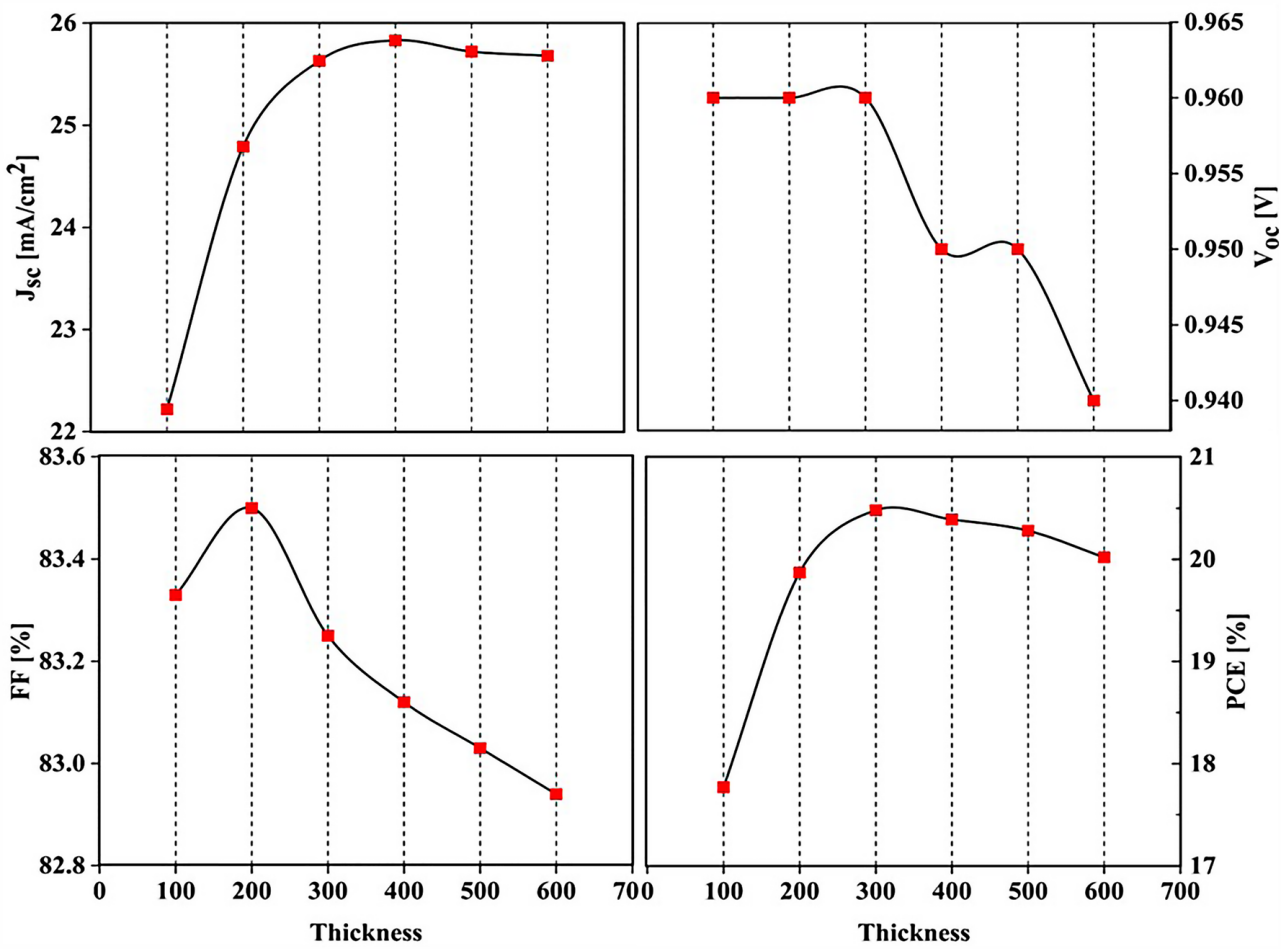
Variation of solar cell performance with different thicknesses of MASnI_3_.

For optimization, we first examined the MASnI_3_ layer from the thickness of 100–600 and put its results in Fig. 18. Performance of solar cells is substantially impacted by the absorber layer's thickness. The performance of PSCs varies depending on the thickness of the MASnI_3_ layer, as seen in Fig. 18. The J_sc_ progressively rises as the MASnI_3_ layer's thickness grows, as shown. But eventually, it thins out to about 500 and 600. However, the V_oc_ is significantly decreased because of an increase in charge recombination in the thicker layer. As a result, when the MASnI_3_ layer becomes thicker, so does the PCE of the PSC. However, the PCE practically achieves its maximum when the thickness approaches 200 nm. Thereafter, the PCE marginally declines. As a result, the maximum PCE in the MASnI_3_ layer's 300 nm thickness is 20.48%.

## Conclusion

This study demonstrates the optical benefits of using the LT structure in PSC. In this work, all PSC layers were designed as LT structures. This LT structure is used to avoid additional costs to increase optical efficiency, and it also has an easy manufacturing process. To improve the optical efficiency of the LT structure, an SiO_2_ layer was used as an anti-reflective layer. This process reduced the reflectance in the cell and increased the PCE from 13.40 (%) to 14.50 (%). Because the planar structure's perovskite (CH_3_NH_3_PbI_3_) material has a band gap of 1.55 eV, the absorption and G_opt_ at wavelengths above 800 nm are close to zero. To solve this problem, CH_3_NH_3_SnI_3_ with a suitable band gap (1.3 eV) was used to increase G_opt_ at wavelengths above 800 nm in the form of a tandem, and for this structure the electrical values of J_sc_ (mA/cm^2^), V_oc_ (V), FF (%), and PCE (%) were equal to 21.97, 0.96, 83.33, and 17.57, respectively. We examined the thickness of the SiO_2_ layer, and the system efficiency improved to 17.77%. In the last part, to get more efficiency, we optimized the MASnI_3_ layer and we were able to reach the highest J_sc_ (22.22 mA/cm^2^), V_oc_ (0.96 V), FF (83.33) and PCE (20.48%). Finally, photon substrates can be used in other types of solar cells with little optimization at no extra cost and improve the light efficiency of solar cells that have light problems.

## Data Availability

The datasets used and/or analyzed during the current study are available from the corresponding author on reasonable request.
